# Tackling Current Biomedical Challenges With Frontier Biofabrication and Organ-On-A-Chip Technologies

**DOI:** 10.3389/fbioe.2021.732130

**Published:** 2021-09-16

**Authors:** Nehar Celikkin, Dario Presutti, Fabio Maiullari, Ersilia Fornetti, Tarun Agarwal, Alessia Paradiso, Marina Volpi, Wojciech Święszkowski, Claudia Bearzi, Andrea Barbetta, Yu Shrike Zhang, Cesare Gargioli, Roberto Rizzi, Marco Costantini

**Affiliations:** ^1^ Institute of Physical Chemistry, Polish Academy of Sciences, Warsaw, Poland; ^2^ Istituto Nazionale Genetica Molecolare INGM “Romeo Ed Enrica Invernizzi”, Milan, Italy; ^3^ Department of Biology, Rome University Tor Vergata, Rome, Italy; ^4^ Department of Biotechnology, Indian Institute of Technology Kharagpur, Kharagpur, India; ^5^ Faculty of Materials Science and Engineering, Warsaw University of Technology, Warsaw, Poland; ^6^ Institute of Genetic and Biomedical Research, National Research Council of Italy (IRGB-CNR), Milan, Italy; ^7^ Department of Chemistry, Sapienza University of Rome, Rome, Italy; ^8^ Division of Engineering in Medicine, Department of Medicine, Brigham and Women’s Hospital and Harvard Medical School, Cambridge, MA, United States; ^9^ Institute of Biomedical Technologies, National Research Council of Italy (ITB-CNR), Milan, Italy

**Keywords:** 3D biofabrication, organ-on-a-chip, tissue engineering, regenerative medicine, precision medicine, drug development

## Abstract

In the last decades, biomedical research has significantly boomed in the academia and industrial sectors, and it is expected to continue to grow at a rapid pace in the future. An in-depth analysis of such growth is not trivial, given the intrinsic multidisciplinary nature of biomedical research. Nevertheless, technological advances are among the main factors which have enabled such progress. In this review, we discuss the contribution of two state-of-the-art technologies–namely biofabrication and organ-on-a-chip–in a selection of biomedical research areas. We start by providing an overview of these technologies and their capacities in fabricating advanced *in vitro* tissue/organ models. We then analyze their impact on addressing a range of current biomedical challenges. Ultimately, we speculate about their future developments by integrating these technologies with other cutting-edge research fields such as artificial intelligence and big data analysis.

## Introduction

At its onset, at the end of the 1980s, the paramount goal of tissue engineering was to manufacture *ex vivo* cellularized substitutes to restore, maintain, or improve tissue functions *in vivo*, which could, ultimately, be used as building blocks for the production of whole functional organs. Although the latter is still almost exclusively the subject of science-fiction stories, researchers in the last three decades have made enormous progress in the fabrication of advanced substitutes capable of recapitulating specific functions of tissues and organs. As a result of these advancements, the application areas of tissue engineering have considerably broadened from the simple manufacturing of tissue substitutes towards being an answer to today’s main biomedical challenges, including drug development, disease, and tissue/organ modeling, and precision medicine. To better understand the progress achieved and the current trends, one should look backward and briefly analyze how the field has been established and evolved, paying particular attention to the technological advances that have been progressively introduced to meet the biological and clinical needs (Hubbell, 1995; Kim et al., 2000; Kaul and Ventikos, 2015).

Originally, tissue engineering found its driving force in the materials science community despite its intrinsic multidisciplinary nature. Pioneers of the field fabricated what can be termed the first generation of tissue-engineered substitutes, mainly aimed at discovering the cross-talk among materials, scaffold architecture, biological cues, and cells (Hollister, 2005; Vallet-Regí et al., 2007; Santos et al., 2010). In particular, the very introduction of three-dimensional (3D) materials for cell culture—generally referred to as scaffolds—generated a great wave of enthusiasm in the field as, for the first time, researchers demonstrated that the *in vitro* cellular responses could be significantly improved compared to standard two-dimensional (2D) cell cultures in plates (Knight and Przyborski, 2015; Souza et al., 2018). In the first studies, researchers had adapted prior existing technologies, such as solvent casting, particulate leaching, freeze-drying, gas foaming, and electrospinning to the manufacturing of biocompatible, highly porous 3D scaffolds (Guarino and Ambrosio, 2010; Raeisdasteh Hokmabad et al., 2017; Parham et al., 2020). 3D cell culture triggered a multitude of studies that originated invaluable information regarding cell attachment cues and cell-cell interactions, cytocompatibility of materials, and most importantly, identified the challenges faced. At the same time, the promising results captured the attention of researchers with a more biological background, laying the basis for the rapid implementation of such new 3D systems within their research pipeline.

**FIGURE 1:**
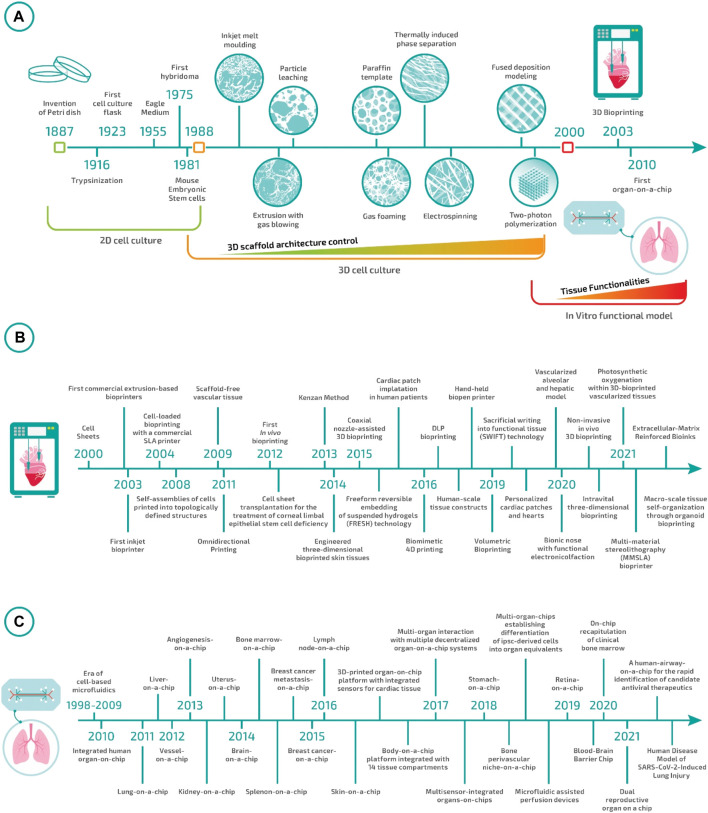
Tissue engineering evolution: **(A)** from Petri dishes to biofabrication and organ-on-a-chip. **(B)** Milestones in bioprinting in the 21^st^ century (Kwon et al., 2000; Landers et al., 2002; Wilson Jr and Boland, 2003; Dhariwala et al., 2004; Jakab et al., 2008; Norotte et al., 2009; Wu et al., 2011; Skardal et al., 2012; Nakayama, 2013; Hinton et al., 2015; Gao et al., 2015; Kang et al., 2016; Retting et al., 2016; D O’Connell et al., 2016; Gladman et al., 2016; Lee J. H. et al., 2017; Pyo et al., 2017, 2017; Bernal et al., 2019; Skylar-Scott et al., 2019; Grigoryan et al., 2019, 2021; Jodat et al., 2020; Chen et al., 2020; Urciuolo et al., 2020; Brassard et al., 2021; Maharjan et al., 2021; Markert et al., 2021; De Santis et al., 2021). **(C)** Milestones in Organ-on-Chip in the 21^st^ century (Huh et al., 2010; Nakao et al., 2011; Franco and Gerhardt, 2012; Jang et al., 2013; Nguyen et al., 2013; Wei-Xuan et al., 2013; Bersini et al., 2014; Rigat-Brugarolas et al., 2014; Torisawa et al., 2014; Choi et al., 2015; Park et al., 2015; Miller and Shuler, 2016; Rosa et al., 2016; Wufuer et al., 2016; Zhang et al., 2017a, 2021; Lind et al., 2017; Vernetti et al., 2017; Wikswo et al., 2017; Ingber et al., 2018; Lee et al., 2018; Marturano-Kruik et al., 2018; Achberger et al., 2019; Park et al., 2019; Chou et al., 2020; Marx and Ramme, 2020; Park et al., 2020; Varghese et al., 2020; Si et al., 2021).

Such a research phase continued for almost a decade until the end of the 1990s. During this time, the field advanced steadily on multiple fronts. In particular, researchers noticed that the methods for scaffold manufacturing were not entirely adequate to ensure neither overall control over macro- and micro-architecture of the 3D scaffolds nor cell distribution throughout them (Figure 1). As a direct consequence of these two issues, the repeatability of the experiments was relatively low, with the impossibility of comparing the results produced in different studies. This ignited the development of a plethora of different techniques for scaffold fabrication and the adaptation/redesign of old methods to the new needs. The peak in controlling scaffold architecture was finally reached at the end of the 1990s with the introduction of the 3D printing technologies (Mironov et al., 2003, 2009; Campbell and Weiss, 2007; Berthiaume et al., 2011). Such technologies enabled the fabrication of pre-determined 3D structures with unprecedented resolution, accuracy, and reproducibility. However, the fabricated 3D materials still required to be seeded with cells, and thus, all the issues connected with cell distribution and overall experiment repeatability were unmet. At the same time, researchers realized that culturing cells in 3D was not enough to attain tissular organization and, in order to move forward, it was essential to guide cells to form a functional tissue—i.e., a macroscopic structure that exhibits a histoarchitecture and functionalities comparable to the target tissue.

At the beginning of this century, a new research era started having as the main focus the development of functional tissue models. This research line rapidly grew due to the increasing evidence of the inadequacy of animal models in predicting human responses, especially regarding drug safety and therapeutic efficacy. The aspiration to develop functional tissue models introduced new challenges to be faced that, in turn, established a new set of technological and biological needs.

Fabricating a macroscopic, 3D cellularized structure with biomimetic features requires precise control at the microscale over the spatial distribution of different cell types, matrix composition, and physicochemical stimuli. Without such control, cells would organize in a quasi-random manner with minimal functionalities and histoarchitectures far from the native ones (Figure 1). In this context, two groups of technologies have recently been established: biofabrication and organ-on-a-chip (Zhang and Radisic, 2017; Moroni et al., 2018a). Biofabrication technologies—which include bioprinting and bioassembly approaches—can be considered an evolution of 3D printing technologies specifically designed for the direct deposition of a bioink—i.e., a physiological solution containing living cells and matrix precursors—or cell-containing building blocks (Groll et al., 2016). On the other hand, organ-on-a-chip systems have been developed as a side branch of microfluidics. These systems comprise micro-channels and chambers where cells can be cultured under extremely controlled conditions, enabling the recapitulation of tissue/organ multi-cellular architectures, tissue-tissue interfaces, physicochemical microenvironments, and vascular perfusion of the body (Bhatia and Ingber, 2014).

In this review, we intend to outline the recent biotechnological advancements obtained using biofabrication and organ-on-a-chip technologies and provide an insight into how these two technologies can be used complementarily to assist biomedical research from bench to bedside.

## Biofabrication vs Organ-On-A-Chip: Characteristic Advantages, Limitations, and Challenges

**FIGURE 2:**
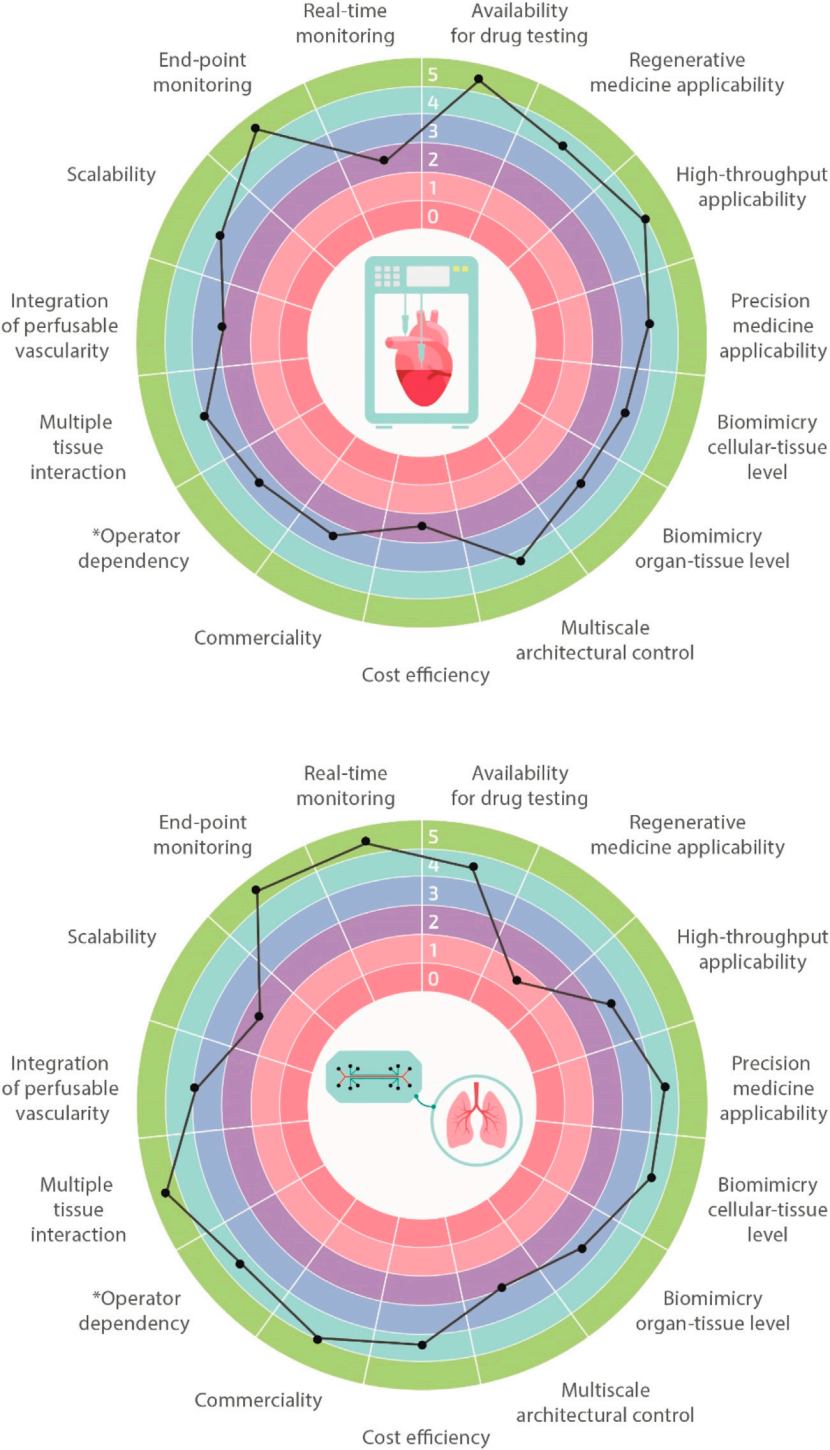
Biofabrication vs organ-on-a-chip. Multi-feature comparisons between biofabrication and organ-on-a-chip technologies highlighting their advantages, limitations, and challenges. Features are classified using a 1–5 scale with 1 meaning very limited and 5 well-suitable (e.g., operator dependency: 1 highly operator dependent, 5 not operator dependent).

Biofabrication and organ-on-a-chip technologies have the potential to revolutionize and boost biomedical research in the next few decades, eventually enabling the development of extremely accurate and functional *in vitro* tissues, organs, and disease models. In order to achieve this ambitious goal, it is paramount to recapitulate the microenvironment and the 3D spatial distribution of cells and extracellular matrix (ECM), ensuring native-like functionality both at the single-cell and tissue/organ levels. Currently, we are still far from fully addressing these challenges, and both biofabrication and organ-on-a-chip technologies suffer from some limits. Nevertheless, given their peculiar advantages, each of these technologies is finding concrete applications in different branches of biomedical research, becoming complementary rather than alternative to one another. Comparison charts of salient features for the two technologies are shown in Figure 2. For instance, biofabrication strategies are very suitable for regenerative medicine purposes through the manufacturing of biological constructs of clinically relevant size characterized by multi-scale, biomimetic architectures. On the other hand, organ-on-a-chip systems represent ideal platforms for higher-throughput studies such as those dealing with drug screening or toxicology. Moreover, they can be employed for studying the impact of various physiological stimuli on the cellular phenotype. In the next two paragraphs, we will provide the readers more insights regarding these two cutting-edge approaches.

### Biofabrication: Building Functional Living Structures in 3D

**FIGURE 3:**
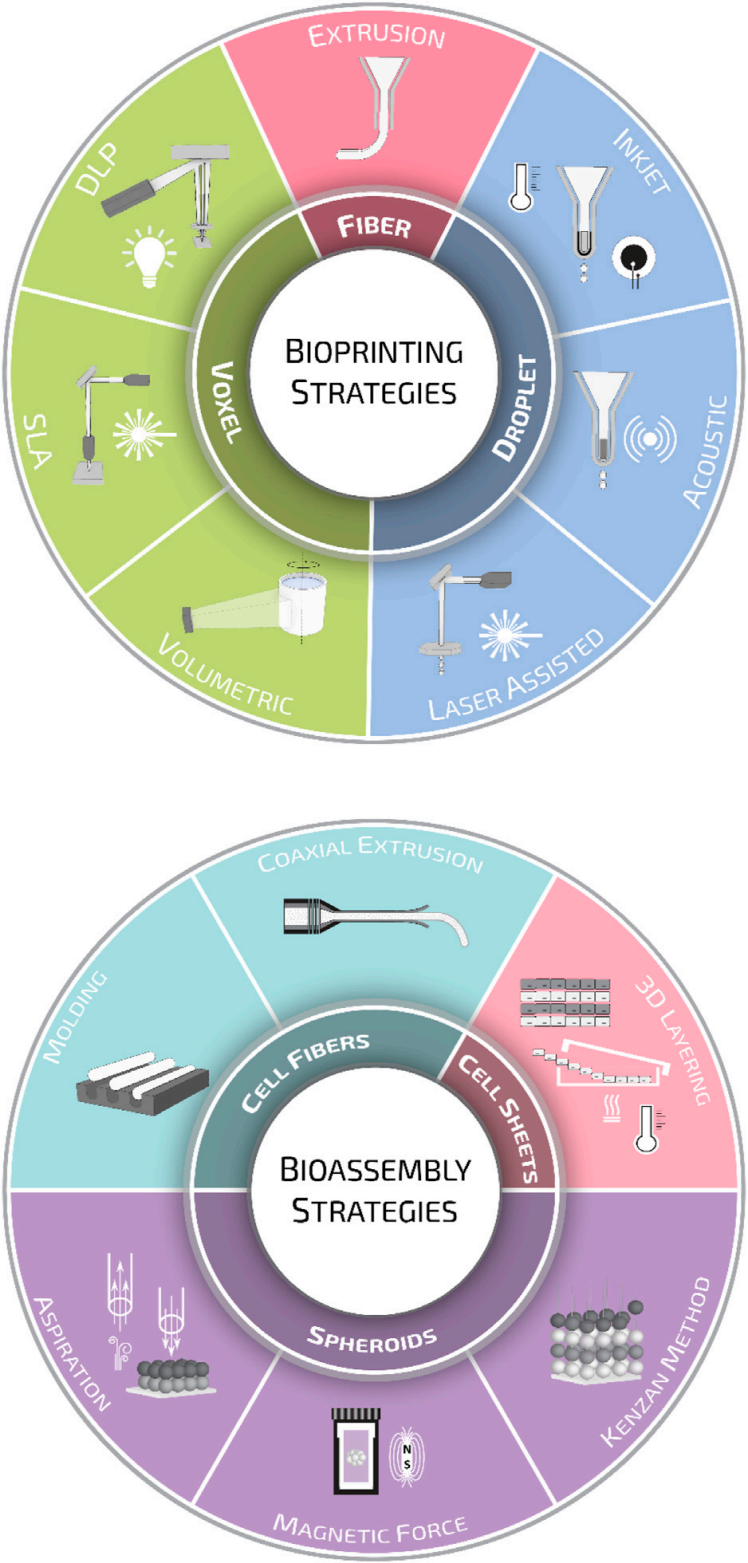
Biofabrication strategies. Schematic representation of the various biofabrication methods employed for scaffold manufacturing divided into bioprinting and bioassembly strategies.

The extensive knowledge and advancements made in the medical, biological, and biotechnological fields have highlighted the limitations of conventional 2D cell cultures in the last decades. The increasing scientific needs demand the design and generation of advanced biomimetic 3D cellular and tissue models. The implementation of 3D manufacturing and materials science in biomedical developments have revealed relatively newer and more complex aspects, such as tissue microenvironments, cell-cell or cell-matrix interactions, and, most importantly, in recapitulating *in vitro* tissue/organ functionality. As anticipated in the introduction, biofabrication technologies have been developed in the last two decades as an extension of the 3D printing strategies for traditional scaffold fabrication. Since then, the field has greatly evolved, and nowadays, the term biofabrication groups together several additive manufacturing strategies. According to a recent publication, biofabrication is defined as the automated generation of biologically functional products with a structural organization from living cells, bioactive molecules, biomaterials, cell aggregates such as micro-tissues or hybrid cell-material constructs through bioprinting or bioassembly and subsequent tissue maturation processes (Groll et al., 2016). Following this definition, biofabrication strategies are primarily divided into bioprinting and bioassembly technologies. While bioprinting enables the 3D spatial arrangement of cells, biomaterials, and biologically active factors, bioassembly facilitates the modular fabrication of bio-structures using cell-containing units (Moroni et al., 2018b; Dalton et al., 2020). However, this primary classification is further ramified, and one can find several other sub-categories for both bioprinting and bioassembly (Figure 3). A thorough description of all these strategies is out of the scope of this review. Nevertheless, to help the readers going through the following sections, we will provide a brief overview of the most common biofabrication approaches, highlighting the differences in terms of cell/material deposition strategies and the quality of bio-substitutes fabricated.

Bioprinting modalities can be sorted into three main groups: droplet-, fiber-, and voxel-based bioprinting (Gudapati et al., 2016; Ozbolat and Hospodiuk, 2016; Hong et al., 2018; Vijayavenkataraman et al., 2018). Droplet-based bioprinting is a non-contact printing process that focuses on the precise sequential positioning of cells in a single bioink drop (Angelopoulos et al., 2020). Droplet formation can be achieved through inkjet or drop-on-demand printing (DOD) (Derby, 2010). In inkjet systems, droplets are generated using thermal (Cui et al., 2010; Solis et al., 2019) or piezoelectric (Hewes et al., 2017; Masaeli et al., 2020) effect; in contrast, in DOD platforms, bioink drops are ejected onto a substrate using a laser beam (Kingsley et al., 2019) or acoustic waves (Foresti et al., 2018; Swaminathan et al., 2019). Fiber-based bioprinting is the most popular bioprinting approach, and in this frame, bioinks are loaded in disposable cartridges and extruded pneumatically or mechanically on sterile substrates. Extrusion systems are mainly based on the generation of continuous hydrogel filaments, which are generally solidified through photocuring (Maiullari et al., 2018), enzymatic (Tijore et al., 2018), ionic, (Kingsley et al., 2019), or thermal (Swaminathan et al., 2019) crosslinking. Lately, thanks to extensive research in the synthesis of photoresponsive biocompatible materials, voxel-based bioprinting systems, such as stereolithography (SLA) and digital light processing (DLP), are becoming increasingly popular. The main difference between these two systems is that SLA systems use a laser beam that moves from point to point to cure the resin, while in DLP systems, the UV light source is stationary, curing a complete layer of resin at a time (Hribar et al., 2014; Miao et al., 2018; Bernal et al., 2019).

The crucial point for bioprinting is choosing the proper bioprinting strategy and designing a biocompatible, instructive, and supportive bioink for the desired application (Gungor-Ozkerim et al., 2018; Ji and Guvendiren, 2021). Gelation kinetics, viscoelastic properties of biomaterial inks, and fabrication time directly influence the resolution, printing fidelity, and cell viability in the bioprinted constructs (Malda et al., 2013). For instance, low-viscosity bioinks are suitable for droplet-based bioprinting as the low-viscosity favors droplet formation at the printhead. On the contrary, high-viscosity bioinks are generally more suitable for fiber-based bioprinting, where the high viscosity of the bioink prevents the breaking and collapse of the deposited filaments during/after printing. Regardless of viscosity, for voxel-based printing, the use of photoresponsive, fast-curing bioinks is crucial to obtain high resolution, cell viability, and shape fidelity.

Alongside 3D bioprinting, bioassembly strategies are the other pillar of biofabrication. Bioassembly is defined as the fabrication of hierarchical constructs with a prescribed 2D or 3D organization through an automated assembly of pre-formed cell-containing units (Groll et al., 2016). One of the most sophisticated bioassembly approaches up to date can be considered as the organoids. These complex and highly trackable constructs are mainly inspired by embryogenic organogenesis, and the research based on organoids unravels the current challenges regarding tissue formation, cell-cell interactions, and tissue functionality (Takebe and Wells, 2019). Bioassembly approaches can be divided as well into three main categories according to the type of cell building block: spheroid-, cell-sheet-, and cell-fibers-based methods (Morimoto et al., 2015).

Strategies based on spheroids rely on the deposition through a nozzle or the assembly using external forces–such as aspiration or magnetic force–of pre-formed spheroids (Tseng et al., 2015; Ayan et al., 2020). Alternatively, pre-formed spheroids can also be assembled in 3D using arrays of tiny needles, a procedure known as the Kenzan method. Upon deposition, adjacent tissue spheroids undergo self-assembly forming larger tissue-engineered constructs (Murata et al., 2020).

**FIGURE 4:**
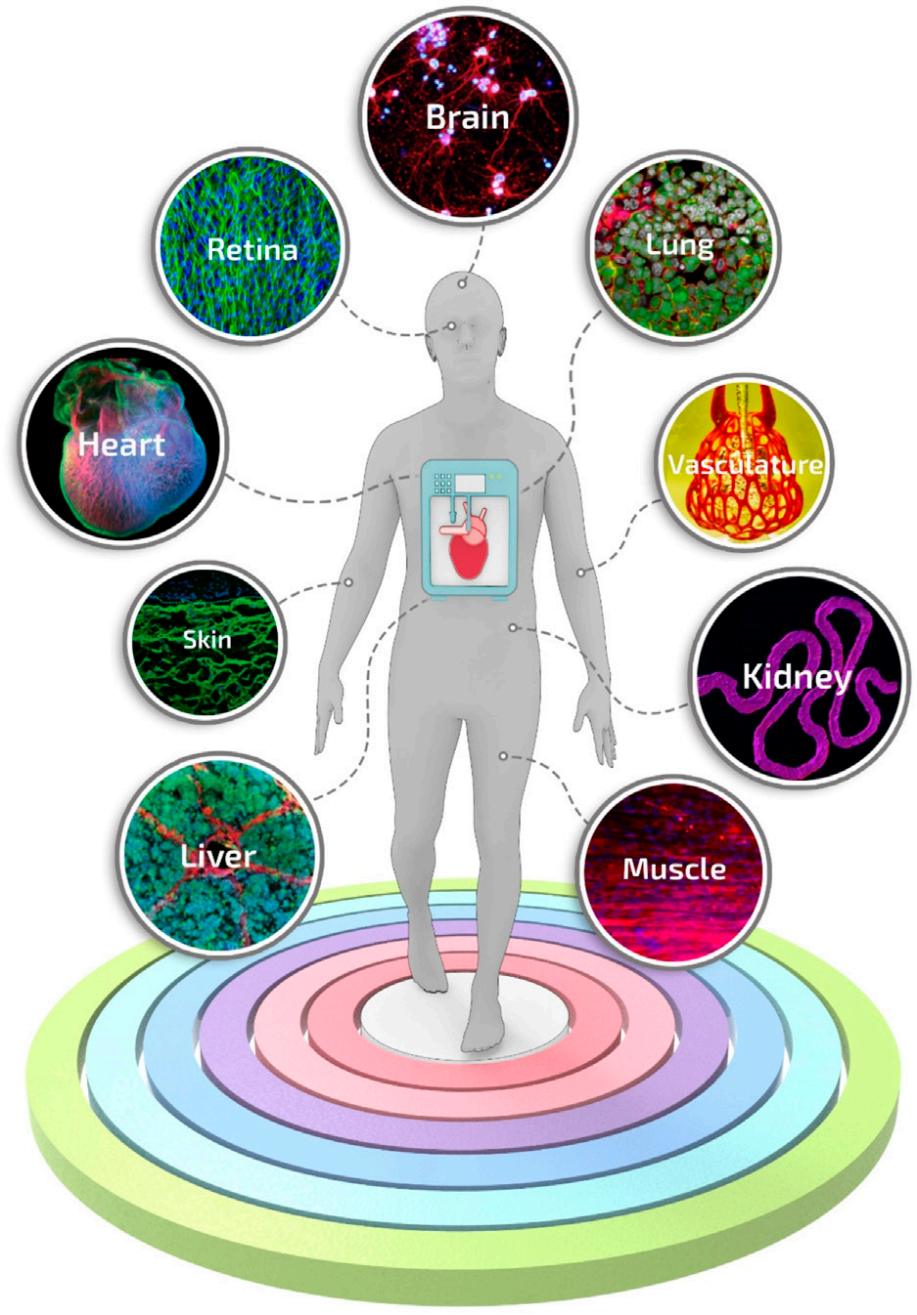
Engineered tissue and organ models manufactured using biofabrication strategies. A selection of representative and advanced studies carried out with biofabrication systems: 3D printing of layered brain-like structures reproduced from ref. (Lozano et al., 2015), hybrid 3D bioprinting of retina reproduced from ref. (Shi et al., 2017), engineering *in vitro* air-blood barrier reproduced from ref. (Horváth et al., 2015), 3D printing of complex biological structures of the heart reproduced from ref. (Hinton et al., 2015), multi vascular networks and functional intravascular topologies reproduced from ref. (Grigoryan et al., 2019), bioprinting of multiscale hepatic lobules reproduced from ref. (Kang et al., 2020), bioprinting of 3D convoluted renal proximal tubules reproduced from ref. (Homan et al., 2016), microfluidic-enhanced 3D bioprinting of aligned myoblast-laden hydrogels reproduced from ref. (Costantini et al., 2017), 3D bioprinting using scaffold-free approach reproduced from ref. (Pourchet et al., 2017).

Besides spheroid constructs, temperature-responsive culture plates (Shimizu et al., 2002; Sekine et al., 2013) have been used to obtain multi-cellular stratified cell-sheet building blocks. The temperature-responsive culture plates have been designed by grafting the polymer poly (N-isopropyl acrylamide) (p-NIPAM) to ordinary tissue culture dishes. Briefly, these temperature-responsive culture plates are relatively hydrophobic and cell-adherent under standard culture conditions. However, upon temperature reduction below the polymer’s lower critical solution temperature, the polymer surface becomes hydrophilic and swells, triggering the spontaneous detachment of the cell layer (Yang et al., 2005). Cell-fiber constructs consist of cell-seeded or cell-laden hydrogel fibers in which cells, after a certain culturing time, organize themselves into densely packed cell cords. For such applications, fibers are generally manufactured by molding (Shimizu et al., 2015) or laminar flow extrusion (Lee et al., 2009; Skylar-Scott et al., 2019; Watanabe et al., 2020). The plethora of biofabrication methods developed so far has generated extensive knowledge, and nowadays, first-generation functional models for several tissues and organs can be manufactured (Figure 4). Nevertheless, these models are in their infancy, still missing some key features needed to introduce disrupting systems in biomedical research truly.

### Organ-On-A-Chip: Tailoring Cell Behavior at the Microscale

Recently, a wave of excitement has spread among the tissue engineering community following the development of new cell culture platforms, the so-called organ-on-a-chip. An organ-on-a-chip is a microfluidic cell culture device that contains continuously perfused and/or actuated (generally negative pressure-actuated) micro-chambers/channels populated by living cells arranged to simulate tissue- and organ-level physiology.

Born from the convergence of various biomedical research fields and microfluidics, organ-on-a-chips have aroused in the last two decades a growing interest in the scientific community for their potential in generating faithful and high-content reproduction of functional tissue units.

Organ-on-a-chips are generally manufactured using conventional soft-lithographic approaches, and, as one could expect, the first-choice material used for their fabrication is polydimethylsiloxane (PDMS) thanks to its moldability, biocompatibility, gas diffusion properties, and optical transparency, the latter representing a key feature enabling live monitoring of cells (Bhagat et al., 2007; Hongbin et al., 2009). More recently, 3D printing technologies—in particular SLA systems—are emerging as an alternative to soft-lithography for the rapid prototyping of organ-on-a-chips (Amin et al., 2016; Park et al., 2018). The reasons for that are numerous, including the reduction of costs associated with organ-on-a-chip manufacturing, ease of use, and the possibility to create more intricate, fully 3D microfluidic devices in a single step.

**FIGURE 5:**
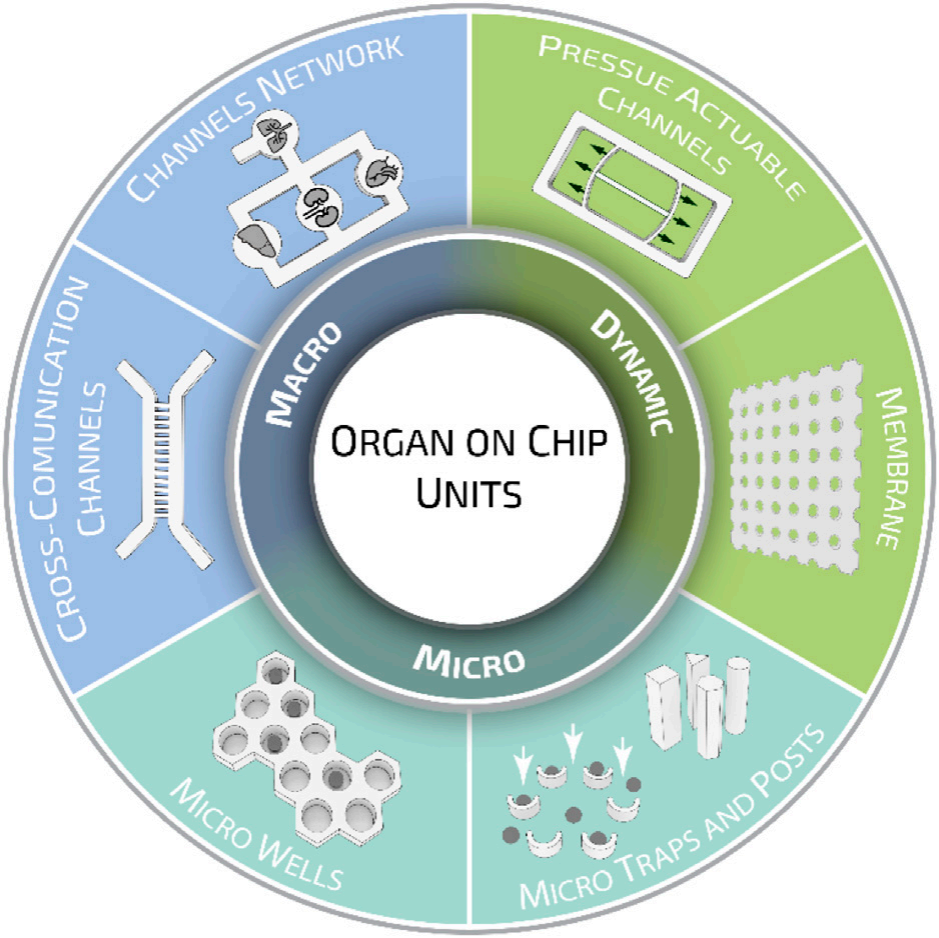
Organ-on-a-chip geometrical units. Different geometrical and structural organ-on-a-chip elements are grouped in three major categories: micro, macro, and dynamic units. While micro units provide stimuli or confined cells at a small cluster or single-cell level, macro and dynamic units are employed to recapitulate specific systemic functions. Such division has been proposed for the sake of presentation, however, organ-on-a-chip units can be designed to possess multiple elements belonging to different groups.

Organ-on-a-chips have been previously categorized for their functions, methods of fabrication, and tissue-specific applications (Ahadian et al., 2018; Wu et al., 2020). Here, we would like to propose a different classification meter based on the geometrical characteristics of organ-on-a-chip units. When analyzed closely, these can be classified into three major categories according to their intended purpose: micro-units, macro units, and dynamic units. Organ-on-a-chip micro units—such as microwells, traps, and posts—are mainly designed to provide stimuli or confined cells at a small cluster or even at the single-cell level. Organ-on-a-chip macro units can be described as channel networks and cross-communication channels, where multiple cell types can reproduce the systemic interaction and response of several tissue models. Dynamic units, instead, indicate all those components that can be actively deformed, inducing tailored stimuli fundamental to study the mechano-responses of cells/tissues under investigation and better recapitulating the actual *in vivo* conditions (Figure 5).

By recapitulating the multi-cellular architectures, tissue-tissue interfaces, physicochemical microenvironments, and vascular perfusion of the body, these devices produce levels of tissue and organ functionality that are not possible to achieve neither with conventional 2D or 3D culture systems nor with animals or human patients. They also enable high-resolution, real-time imaging and *in vitro* analysis of biochemical, genetic, and metabolic activities of living cells in a functional tissue- and organ context (Jackson and Lu, 2016). Moreover, organ-on-a-chip microsystems are particularly suitable for higher throughput screening, such as drug testing. Nowadays, the latter application is particularly under exploitation. If successful, it should be especially valuable for studying molecular mechanisms of action, prioritization of lead candidates, toxicity testing, and biomarker identification (Paul et al., 2010).

**FIGURE 6:**
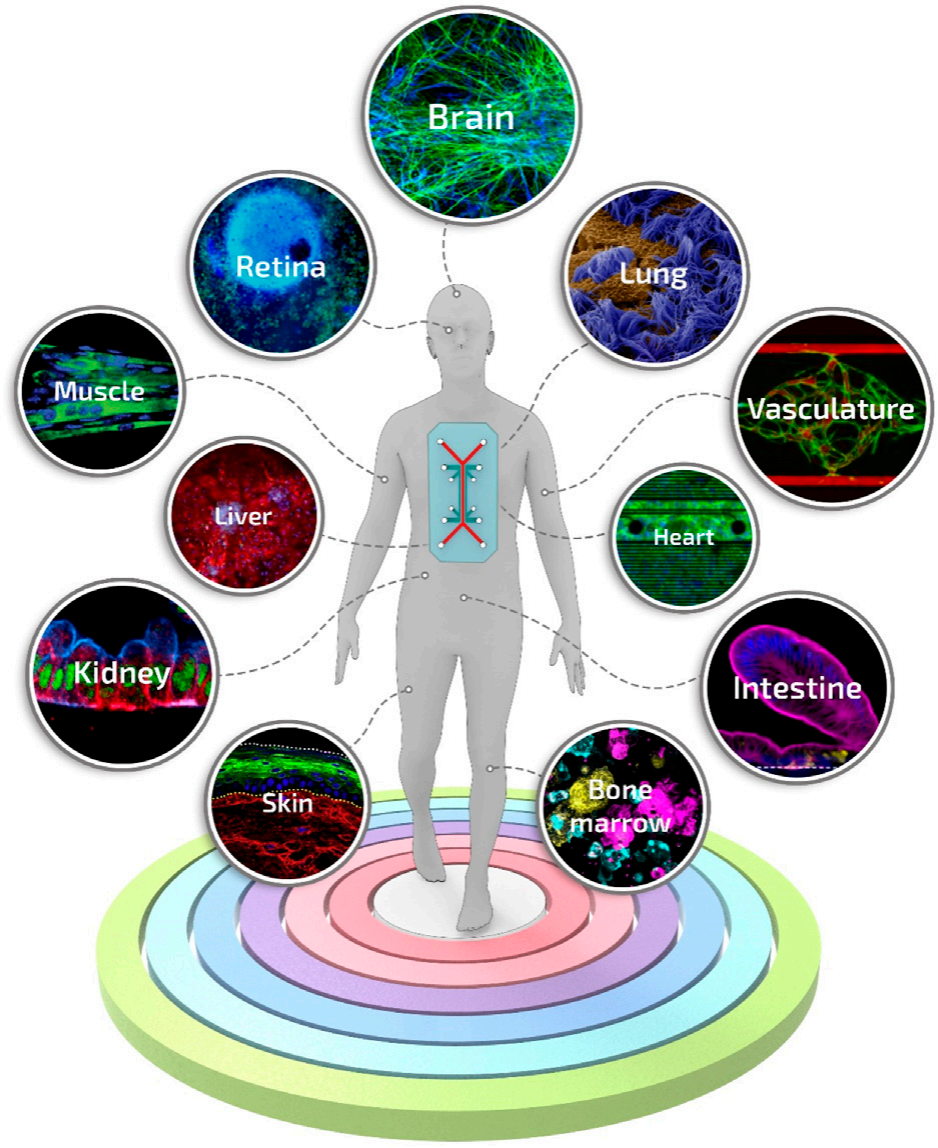
Engineered tissues and organs manufactured using the organ-on-a-chip technology. A selection of the representative and advanced studies carried out with organ-on-a-chip systems: blood-brain-barrier-on-a-chip reproduced from ref. (Vatine et al., 2019), retina-on-a-chip reproduced from ref. (Achberger et al., 2019), small airway-on-a-chip reproduced from ref. (Benam et al., 2016), liver-on-a-chip reproduced from ref. (Jang et al., 2019), vascularized and perfused organ-on-a-chip platform reproduced from ref. (Phan et al., 2017), tubuloids of human adult kidney-on-a-chip reproduced from ref. (Schutgens et al., 2019), heart-on-a-chip reproduced from ref. (Qian et al., 2017), skeletal muscle-on-a-chip reproduced from ref. (Agrawal et al., 2017), small intestine-on-a-chip reproduced from ref. (Kasendra et al., 2018), full-thickness human skin-on-a-chip reproduced from ref. (Sriram et al., 2018), on-chip recapitulation of clinical bone marrow reproduced from ref. (Chou et al., 2020).

To date, a great variety of organs and tissues have been recapitulated into organ-on-a-chip-based systems, including liver (Nakao et al., 2011), kidney (Jang and Suh, 2010), intestine (Kim et al., 2016), lung (Douville et al., 2011), heart (Grosberg et al., 2011), muscle (Grosberg et al., 2012), vessel (Song and Munn, 2011), and bone marrow (Torisawa et al., 2014), up to complex models of body-on-a-chip (Esch et al., 2011) (Figure 6). However, despite the critical breakthroughs achieved in the field, a pressing unmet question that continues to emerge is the data-supported evidence of its actual benefits compared to existing conventional models or well-established tissue engineering approaches (Mastrangeli et al., 2019b). For instance, the advantages of the inclusion of dynamic perfusion and *in situ* stimulation in organ-on-a-chip are becoming evident only recently and more substantial readout correlations with clinical and human physiological behavior are still needed (Del Rio et al., 2017; Vernetti et al., 2017).

## Tackling Current Biomedical Challenges With Frontier Technologies

Since their establishment, biofabrication and organ-on-a-chip technologies have evolved notably and nowadays represent the most promising approaches to overcome the restrictions of conventional biological and medical research methods. Together with these techniques, biomedical research has greatly advanced and branched into a multitude of sub-fields that reflect its intrinsic, complex heterogeneity. In this context, biofabrication and organ-on-a-chips are now called to face and possibly address relevant biomedical challenges such as 1) the manufacturing of spare tissues/organs to fight the shortage of organs’ donors, 2) the fabrication of reliable physiological and pathophysiological *in vitro* tissue/organ models to replace animal testing and to unravel new disease markers and mechanisms, 3) the reduction of costs connected with new drug development, and 4) the formulation of precision medicine therapeutic protocols. Indeed, these are long-term challenges, and still, many years will pass before one could actually solve them. Nevertheless, biofabrication (Table 1) and organ-on-a-chips systems (Table 2) have already contributed and, more importantly, are expected to further contribute significantly in the near future to address them. Below, we will briefly report and analyze the impact of these technologies in a selection of relevant biomedical challenges, including tissue engineering and regenerative medicine, disease models for precision medicine, drug development and testing drug kinetics, and in the growing field of omics.

**TABLE 1:** Representative studies based on biofabrication technologies reported for the selected biomedical applications.

Target organ/tissue	Biomedical application	Main findings	Ref
Cardiac Tissue	Regenerative medicine	Generation of complex 3D biological structures using soft hydrogels	Hinton et al. (2015)
		3D-bioprinted human cardiomyocytes showing synchronized contractions and directional action potential propagation	Lee et al. (2019a)
		Heterogeneous constructs containing endothelial cells and iPSC-derived cardiomyocytes and effective generation of vessel-like structure with a lumen of approximately 150 μm	Maiullari et al. (2018)
Kidney	Disease modeling and drug screening	3D-perfusable proximal tubules embedded within an engineered ECM with active reabsorption of solutes *via* tubular–vascular exchange	Homan et al. (2016)
		Integration of adjacent open lumens embedded within a permeable ECM, confluent endothelium and epithelium that circumscribe these lumens, and a closed-loop perfusion system that enhances cell maturity for kidney function	Lin et al. (2019)
	Regenerative medicine	Bioprinted human kidney cells maintaining kidney-specific phenotype and formation of tubular and glomerular-like structures in the construct	Ali et al. (2019)
Liver	Regenerative medicine	Highly vascularized micro- and macro-scale engineered hepatic lobules	Kang et al. (2020)
		Functional hepatic lobules able to generate human liver tissues that could be transplanted	Yang et al. (2020)
Lung	Disease modeling and drug screening	Establishment of an innovative bioprinting tool to engineer an advanced 3D lung model for high-throughput screening	Horváth et al. (2015)
	Regenerative medicine	A “breathing model” with tidal air ventilation and blood flow to demonstrating pulmonary transport by measuring blood oxygenation	Grigoryan et al. (2019)
Skeletal muscle	Regenerative medicine	Fabrication of functional skeletal muscle tissue based on an innovative co-axial needle extrusion 3D bioprinting approach useful for human clinical application	Costantini et al. (2017)
		Skeletal muscle bundle-like structure with highly organized architecture, providing the structural maturation and force-generating capacity of neonatal rat myogenic cells	Kim et al. (2018)
		Integration of neural cell component for long-term cell survival, to enhance myogenic differentiation, and to induce NMJ formation on the *in vitro*-bioprinted skeletal muscle constructs, rapid restoration of muscle function *in vivo*	Kim et al. (2020)
Neuronal network	Disease modeling	3D bioprinting of brain-like structures that reproduce neuronal architecture to study brain injuries and neurodegenerative diseases	Lozano et al. (2015)
		The first proof-of-concept bioprinted model of the tumor microenvironment that displays macrophage recruitment and polarization as well as glioblastoma progression and invasion in a single construct	Heinrich et al. (2019)
		3D bioprinting of 3D brain-like co-culture construct composing heterogenous neural populations with neurospheroids and glia	Li et al. (2020b)
Retina	Disease modeling	Development of a reasonable *in vitro* retina model for studying sight-threatening diseases	Masaeli et al. (2020)
	Regenerative medicine	3D-bioprinting and co-differentiation of retinal progenitor cells (RPCs) into photoreceptors with the support of retinal-pigment epithelium (RPEs) to mimic the native environment during retinal development	Wang et al. (2018b)
Skin	Regenerative medicine	Setup of the first scaffold-free bioprinting strategy for the generation of a full-thickness skin	Pourchet et al. (2017)
		A perfusable 3D skin equivalent composed of hypodermis, dermis, and epidermis	Kim et al. (2019)
		Indistinguishable bilayered dermo-epidermal equivalents generation during *in vivo* skin regeneration	Cubo et al. (2016)
Vasculature	Regenerative medicine	Identification of a stereolithographic process for simultaneous and orthogonal control over tissue architecture and biomaterials	Grigoryan et al. (2019)
		Deposition of perfusable vascular structures in highly ordered arrangements through a multilayered coaxial extrusion system in a single-step process	Jia et al. (2016)
		After *in vivo* transplantation, observation of anastomosis between the bioprinted endothelial network and host circulation with functional blood vessels featuring red blood cells	Zhu et al. (2017)

**TABLE 2:** Representative studies based on organ-on-a-chip technologies reported for the selected biomedical applications.

Target organ/tissue	Biomedical application	Main findings	Ref
Neuronal network	Disease modeling and drug screening	Creation of a neurovascular unit that recapitulates complex BBB functions for *in vitro* study of neurological disorders and drug screening	Vatine et al. (2019)
		Observation of abnormal neurite outgrowth, neuronal differentiation, and migration in nicotine-treated brain organoids in brain organoid-on-a-chip system derived from human iPSCs allowing to model neurodevelopmental disorders under prenatal nicotine exposure (PNE) at early stages	Wang et al. (2018d)
		Development of *in vitro* BBB model to evaluate the penetration of large molecules and antibodies	Wevers et al. (2018)
Bone marrow	Disease modeling and drug screening	Fabrication of a microfluidic human bone marrow (BM) chip that mimics key aspects of human hematopoiesis and bone marrow dysfunction	Chou et al. (2020)
		Microfluidic device for the culture of living bone marrow with a functional hematopoietic niche *in vitro*	Torisawa et al. (2014)
		Identification and quantification of preferential interactions of circulating normal and malignant hematopoietic stem cells with distinct niches	Aleman et al. (2019)
Cardiac muscle	Disease modeling	Providing a functional 3D cardiac model for mechanical and biochemical co-stimulation to predict signs of hypertrophic changes in cardiac phenotype	Marsano et al. (2016)
		Development of an engineered fibrosis-induced heart failure model and validation of the model through anti-fibrotic drug treatment	Mastikhina et al. (2020)
		Angiotensin II (ANG II)-induced cardiac dysfunction model to elicit pathological responses in a heart-on-a-chip platform	Horton et al. (2016)
Intestine	Disease modeling and drug screening	Generation of a tool for the investigation of intestine metabolism, nutrition, infection, and drug pharmacokinetics	Kasendra et al. (2018)
		Development of a complex living human gut microbiome, including obligate anaerobes in direct contact with human intestinal epithelial cells and their overlaying mucus layer	Jalili-Firoozinezhad et al. (2019)
		Robust and reliable high throughput gut-on-a-chip model to mimic the key aspects of inflammatory bowel disease pathogenesis	Beaurivage et al. (2019)
Kidney	Disease modeling and Drug screening	Tubuloid-on-a-chip platform as model infectious, malignant, and hereditary kidney diseases for personalized medicine	Schutgens et al. (2019)
		Design of pseudorabies virus (PrV) induced kidney disease model on-chip for identifying disease biomarkers	Wang et al. (2019a)
		An organ-on-a-chip platform to study the pathophysiology of glomerular diseases regarding changes in the 3D conformation of podocytes, endothelial cells, and glomerular basement membrane, abnormalities in their function; and crosstalk among them	Petrosyan et al. (2019)
Liver	Drug screening	Fabrication of a tool that integrates liver cells, liver decellularized ECM, and vascular/biliary fluidic channels	Lee et al. (2019b)
		Liver sinusoid-on-a-chip model and uniform distribution of discrete HUVECs	Mi et al. (2018b)
		*In vitro* development of non-alcoholic fatty liver disease and observation dependency of diet on the reversibility of steatosis	Lasli et al. (2019)
Lung	Disease modeling and drug screening	Development of chip for mimic human lung inflammatory disorders to detect synergistic effects of lung endothelium and epithelium on cytokine secretion and identify new disease targets	Benam et al. (2016)
		Enhancement of lung-on-a-chip microdevice with a nanofiber membrane for human non-small cell lung cancer anti-cancer drug testing	Yang et al. (2018)
		Design of a murine lung-on-a-chip *Mycobacterium tuberculosis* model to reveal the dynamics in the host at an air-liquid interface with a spatiotemporal resolution	Thacker et al. (2020)
Skeletal muscle	Disease modeling and drug screening	Advance skeletal muscle-on-a-chip tool for preclinical drug discovery and development	Agrawal et al. (2017)
		Development of Duchenne muscular dystrophy model-on-a-chip and evaluation of the cell therapy treatment	Serena et al. (2016)
		Evaluation of dynamic drug response, to monitor the contractile force of human skeletal muscle myobundles over time, before and after treatment with drugs	Zhang et al. (2018)
Retina	Drug screening	Development of retina-on-chip for drug testing	Achberger et al. (2019)
		Organotypic eye-on-a-chip model mimicking the retinal pigment epithelium choroid complex *in vitro* with adjacent perfusable blood vessel network	Chung et al. (2018)
Skin	Disease modeling and drug screening	Scalable skin-on-a-chip system for high throughput drug screening and toxicological applications	Sriram et al. (2018)
		Three layer - epidermal, dermal and endothelial - skin-on-a-chip model simulating inflammation, oedema, and drug-based treatment	Wufuer et al. (2016)
Vasculature	Drug screening	Creation of a vascularized micro-organs platform to assay a small library of compounds	Phan et al. (2017)
		A microfluidic tumor-vasculature-on-a-chip model with tumor leaky vasculature and 3D tumor tissue with dense ECM to study extravasation through leaky vasculature and accumulation	Wang et al. (2018a)
		Development of built-in vasculature system for direct surgical anastomosis	Zhang et al. (2016)
Multi-organ-on-a-chip	Disease modelling and drug screening	Heart/liver/cancer-on-a-chip platform for the investigation of the delivery and side effects of drugs metabolites. The research opens the door towards the generation of a “body-on-a-chip”	Kamei et al. (2017)
	Drug screening and omics analysis	Microfluidic liver and kidney platform coupled with nuclear magnetic resonance (NMR)-based metabolomic foot-printing for small-molecule screening approach	Shintu et al. (2012)
		First example of combining human-on-a-chip platform, drug metabolism and metabolomics to investigate complex human physiology and multiorgan interactions	Wang et al. (2019b)

### Tissue Engineering and Regenerative Medicine

Organ and tissue failures due to chronic or genetic diseases, trauma, or infection, represent a major medical issue worldwide, which nowadays can be effectively treated exclusively through organ transplantation. Such surgical operations are extremely invasive, and the placement of a heterologous organ often triggers a severe immune response in the host that causes organ rejection. TERM—two interdisciplinary research fields that combine engineering and life science principles—were proposed during the 1990s to replace, repair, or regenerate human tissues or organs with the ultimate goal of reestablishing normal tissue functions (Langer and Vacanti, 1993).

In the last decade, 3D biofabrication techniques and organ-on-a-chip have served to this goal as complementary approaches. Whereas organ-on-a-chip methodologies have generally provided some inputs to TERM at the cellular level (i.e., micro-tissue level), biofabrication technologies have been successfully tested for the fabrication of larger-scale constructs up to, in few cases, human-sized organs (Kang et al., 2016; Mirdamadi et al., 2020). However, the biofabricated structures, despite their realistic shapes and dimensions, exhibit very limited functionalities, thus being unsuitable directly for clinical applications.

As one could easily infer, fabricating artificial organs is an extremely complex task, and, up to date, it is still difficult to precisely identify and foresee all the actions that may be required for this process. Nevertheless, few fundamental steps will be needed, including 1) dedicated protocols for stem cell identification and expansion, 2) definition of blueprints for target organs/tissues—i.e., complete sets of instructions for automated assembly of the artificial organ/tissue, 3) development of post-printing protocols for engineered organ maturation. Below, we provide a concise description of these three key points and the challenges behind them.

#### Living Matter Matters: Towards Cell Identification and Expansion

Manufacturing engineered autologous tissues require billions to trillions of cells. At the cellular level, the living matter of an engineered tissue should have two intrinsic characteristics, cellular diversity—i.e., the range of different cell types that constitute a tissue—and self-renewal—i.e., perpetuating the cell pool throughout life. Thus, selecting a sustainable, appropriate autologous cell source as the starting point and establishing standardized scaled-up cell expansion processes represent pivotal milestones. Currently, stem cells, including embryonic stem cells (ESCs), adult stem cells (AdSCs), and induced pluripotent stem cells (iPSCs), are the sole remedy to guarantee cellular diversity and self-renewal for TERM applications as terminally differentiated cells tend to lose their proliferative and self-renewal potential rapidly when intensively expanded *in vitro* (Brown et al., 2013).

On the contrary, stem cells can undergo asymmetric divisions—i.e., one stem cell can divide into a progenitor daughter cell while the other one stays in a self-renewal state. Whereas self-renewal of stem cells is significantly advantageous in populating engineered tissue constructs, this phenomenon can jeopardize the sustainability of stem cell expansion processes (Shahriyari and Komarova, 2013). Hence, uncontrolled proliferation and differentiation of stem cells have to be strictly monitored during the expansion, and their phenotypic stability should be certified. Consequently, scaled-up expansion of stem cells would require substantial advancements in the media recipes, culturing devices, and techniques. One way to stabilize these processes can be the automation of the main cell culture variables. Alternatively, new culture conditions and media recipes could guarantee stable expansion, preserving the self-renewal capability and phenotypic stability of stem cells.

#### Deciphering Anatomical Architectural Complexity: Towards Organ Blueprints

The process of designing a blueprint starts with deciphering the anatomical architectural complexity. Currently, the macro architecture of patient-specific tissue substitutes can be unprecedentedly designed using computed tomography (CT) and magnetic resonance imaging (MRI) data (Hosny et al., 2018). However, achieving biomimetic matrix composition and cellular-level complexity of the intact human organs is a daunting task, at first due to the lack of scalable technologies to image human organs at the cellular level. Although the standard histological techniques provide invaluable information of 2D sections, creating a 3D reconstruction of a whole organ from 2D histological sections could be very complicated or impossible because of the numerous tissue section distortions, besides being extremely time-consuming. In this context, some innovative approaches have recently been proposed to better decipher the anatomical complexity of matrix composition and diversity of cell populations (Menden et al., 2020; Zhao et al., 2020). One of these approaches focuses on developing deconvolution algorithms that can estimate different cell fractions and infer cell type-specific gene expressions in a tissue sample (Menden et al., 2020). These algorithms present valuable information regarding cell diversity. However, these algorithms do not provide any information about the spatial distribution of such cell populations. The latter information can be attained using a complementary method based on a 3D deep tissue immunofluorescence staining strategy recently introduced. This method—named SHANEL—involves clearing the tissue with a zwitterionic detergent (CHAPS (3-[(3-cholami- dopropyl)dimethylammonio]-1-propanesulfonate) and then labeling it with a 3D deep-tissue antibody-staining protocol, attaining precise 3D spatial information of specific cell populations (Zhao et al., 2020).

Alongside these prescient studies to resolve the limitations in large organ design, significant success has been achieved by implementing the bottom-up tissue engineering approach in the manufacturing of tissue/organ building blocks—i.e., miniaturized constructs generally having a volume up to 1 cm^3^—which exhibit histoarchitectures and functions close to the native targeted tissue/organ. For instance, researchers have recently succeeded, to some degrees, in fabricating tissue-specific building blocks by recapitulating the architectures and functions of the human liver lobules (Ma et al., 2016; Gori et al., 2020), the dynamic microenvironment of the alveolar-capillary unit (Huh, 2015), the parallel organization of myofiber bundles (Costantini et al., 2021), and the primary human small intestine (Kasendra et al., 2018; Shin et al., 2019). Despite the diversity in their structural and cellular complexity, the conjoined outcomes of these studies indicate that effective architectural guidance provides crosstalk among the cell populations and enhances the morphogenesis of the desired tissue. The preferential alignment of cells through contact guidance along with the topographical features actively impacts polarization, aggregation, and, as a result, differentiation and maturation of the cells. Consequently, in defined architectures, cells can sense organ size and functionality, initiating a cross-talk to terminally differentiate, stop proliferation, and yet, retain a minimal level of stemness for homeostasis and potential for regeneration (dos Santos and Liberali, 2019).

#### Post-Processing of Biofabricated Organs: From Bioreactors to Bedside

Housing billions to trillions of stem cells in anatomically legit architectures and directing them into full-size, functional engineered organs require standardized differentiation protocols and dynamic microenvironments. Inspired from the industrial bioprocesses, bioreactors have been adapted to TERM applications for subjecting 3D engineered tissues to various physiologically relevant stimuli, such as mechanical stresses, nutrient/cytokine gradients, and culturing conditions. An interplay between these physiological stimuli and the cellular components is the key element for ensuring their maturation into full-size, functional engineered organs. As nutrients, oxygen, and their transport mechanisms hold paramount importance for the development of tissue structures and functions, up to date, different bioreactor designs with circulatory system approximation have been proposed to address the difficulties for sufficient nutrient/oxygen supplies through engineered tissue building blocks (Kasendra et al., 2018). Alongside the sufficient nutrient supplies, such reactors have also enhanced tissue maturation as a result of the creating gradients of growth factors, cytokines, changing ECM directionality, and effective degradation of the matrix.

Although these bioreactors can satisfy the needs of long-term cultures in certain aspects, the nutrient diffusion through full organ thickness is still limited without a functional vasculature network. Introducing endothelial cells and a mechanism of angiogenic growth factor release are the current strategies to induce vascularization in engineered tissues. Nevertheless, creating fully vascularized constructs with capillary-size, perfusable, tubular constructs is significantly demanding mainly due to the limited resolution of current biofabrication techniques alongside the extensive phenotypic and zonal heterogeneity of endothelial cells. While various bioreactors have been designed to improve diffusion or vascularization, some bioreactor layouts have also evolved towards introducing physiological stimulation to tissue constructs. In long-term tissue culture, the dynamic state is favorable as it introduces a degree of stress that impacts the performance of engineered organs (Selden and Fuller, 2018). The applied mechanical stresses influence the mechano-sensing properties of the cytoskeleton proteins that play a critical role in representing physiological stimulation effects on cell behaviors. As an example, smooth muscle—endothelial cell co-cultures may transform into arteries-like functionality when subjected to blood pressure loads. However, these co-cultures may exhibit heart valve-like functionality when they are subjected to alternating pressure gradients (Ayoub et al., 2016). Regarding musculoskeletal tissues, bioreactors designed to mimic mechanical stretching and electrical stimulation will provide a good model for studying the functional maturation of tissue constructs (Parrish et al., 2019). As physiological stimulation is required to sustain homeostasis, cell polarity, plasticity, and tissue maturation, surely, at different stages of development, engineered tissues might require different regimes of physiological conditioning due to the maturation of the engineered tissue, changes in the cell population densities and the accumulation of extracellular matrix.

### Disease Models for Precision Medicine

In medicine, signs and symptoms are the core of the diagnostic approach to specify and explain the disease state as well as to establish a prognosis and a therapeutic approach. Nevertheless, the outcome may vary among the patient groups due to their genetic, phenotypical, and psychosocial characteristics. Hence, the recent clinical data on individual patient differences and the developments in biomedical research have propounded the idea of precision medicine. Precision medicine mainly focuses on the treatments that distinguish the individual patients’ needs based on their genetic, clinical, phenotypic, psychosocial idiosyncrasy from other patients with similar clinical representation. The precision medicine hypothesis resides on the premise that stratified disease subgroups can be better treated through precise and validated genetic and phenotypic recognition (Ashley, 2016). There is no steady-state endpoint where precise medical care is provided to the patients; hence, each stratification can be just the interim result of the whole process. The cycles in stratification imply that the prognosis and treatments in patient subgroups become more and more effective with ongoing efforts. For instance, the discovery of CYP2C19 gene’s role in some drug cardiotoxicity—e.g., Terodiline, Clopidogrel, Desipramine—and specific drug efficacies for cancer patients with certain mutations—such as the case of effective taxane chemotherapy for BRCA1 mutation breast cancer patient subgroup—can be considered as some distinctive victories of precision medicine (Wang et al., 2013; Asif et al., 2016; Balasubramaniam et al., 2017). Indeed, precision medicine is not a need or a unique tool for all diseases. Currently, precision medicine is finding promising applications in some medical fields, such as cancer, neurodegenerative and rare diseases, to assure effective treatments and bench-to-bedside transition of these treatments for patient subgroups with similar epigenetic profiles. Despite the major premise of precision medicine, the field requires enhancements in two main aspects for its clinical translation. Specifically, collecting and analyzing the data to stratify the patient subgroups is considered the first main hurdle of precision medicine. Currently, cluster analysis of differently expressed genes is being used to define the patient subgroups as well as to discover the underlying pathological mechanism and a potential target for therapeutic development. Nonetheless, this technique requires full data sets on the genetic, clinical, phenotypic, psychosocial idiosyncrasies of the patients, which are not always obtainable (Kaur and Chupp, 2019). Moreover, handling the out-of-range data that are below or above the detection or threshold limits also restricts the success of cluster analysis. The second aspect to be improved consists of how well the patient subgroups respond to the proposed treatment. Longitudinal assessments—i.e., short-term assessments of treatment efficacy, recurring assessments over a period of time to reinforce treatments, iterative disease remission testing, and follow-up assessments to ensure successful treatment—reflecting the disease courses and planning safe clinical trials are substantial for precision medicine’s clinical translation. Since there is a gap between the data collection and establishing the clinical translation of the targeted treatments, 3D engineered tissue models, and disease-on-a-chip platforms have been proposed to bridge this gap. The use of engineered tissues and disease-on-a-chip can offer new platforms that can model the molecular and cellular disease phenotypes, develop correlated data for the cluster analysis and present longitudinal assessments of therapeutic responses on the bench for clinical translation.

#### Cancer

Cancer is an epigenetic disease caused by mutations to genes that control the way our cells function, especially how they grow and divide. The epigenetic changes that cause cancer can be inherited or can arise during a person’s lifetime due to damage to DNA caused by specific environmental exposures. Over the years, the clinical findings have indicated genetic differences among cancer patients with the same diagnosis. Thus, precision medicine has gained increasing interest, potentially being the next frontier in cancer research (Friedman et al., 2015). Up to date, patient subgroups with non-small cell lung cancers due to the mutation in the anaplastic lymphoma kinase (ALK), BRCA mutant-ovary cancer, breast cancer with human epidermal growth factor receptor 2 (HER2) overexpression have already benefited from precision medicine with targeted therapies (Vogel et al., 2002; Neff et al., 2017; Qin and Gadgeel, 2017).

Nevertheless, clinical translation of precision medicine in cancer research is often hampered due to the cost of genomic testing, inadequate phenotypic and psychosocial patient information, and lack of longitudinal assessment of therapeutic responses. In this frame, biofabricated 3D models and tumor-on-a-chip platforms have been proposed for understanding the cancer progression and efficient bench-to-bed translation of precision medicine. Whereas 3D biofabrication strategies have been able to recapitulate the tumor microenvironment complexity by patterning tissue-specific bioinks and fabricate physiologically relevant tissue constructs with high spatiotemporal control over the 3D structures, tumor-on-a-chip platforms mostly aim to recreate significant features of the tumor physiology at molecular and cellular levels (Yesil-Celiktas et al., 2018). For instance, tumor-on-a-chip platforms have provided unique microenvironments to simulate the overall tumor pathologies (Wang Z. et al., 2019; Wei et al., 2019; Mencattini et al., 2020). In this scenario, single-cell tumor-on-a-chip platforms have been employed to study the complex composition of tumors. These platforms have been used to visualize rare cancer cells in bulk tumors, with the final aim to interpret tumor heterogeneity comprehensively, detect circulating molecules, and widen the current know-how of cancer genomics (Saadatpour et al., 2015; Tsoucas and Yuan, 2017).

Conversely, multiple-cell-type tumor-on-a-chip platforms (e.g., cancer, endothelial, stromal, and immune cells) have been used to investigate the specific crosstalk between different cell populations in the tumor microenvironments such as signaling cascades, tumor angiogenesis, and metastasis pathways (Kolesky et al., 2014; Grolman et al., 2015; Sharifi et al., 2020; Cabon et al., 2021). Alternatively, 3D-bioprinted compartmented multiple-cell constructs have been used to create paracrine loops for studying cancer cell extravasation into the bloodstream and analyze metastatic progression through trans-endothelial migration (Kolesky et al., 2014; Wang et al., 2018c; Li J. et al., 2020). Without a doubt, both the 3D biofabrication and the tumor-on-a-chip platforms have advanced the *in vitro* cancer research up to a new level by providing stable longitudinal real-time monitoring, as well as dynamic microenvironment to discover cancer and metastasis mechanisms which can be used in precision medicine for stratifying sub-groups and planning safe therapeutic strategies for patients. Most likely, these platforms will progressively become key tools for precision medicine application in cancer research in the near future, possibly with the convergence of the inputs from other fields, such as omics, systems biology, and bioinformatics.

#### Rare Diseases

A rare disease is defined as a condition that affects fewer than 200,000 people globally. The major problems associated with rare diseases are the lack of understanding of the disease mechanisms and the very small patient population that prohibits efficient clinical trials to identify effective therapeutic approaches. These issues make rare diseases a focus point of precision medicine. Recently, with the development of iPSCs and CRISPR technologies, establishing relevant mutations on patient-specific cells has become a common practice that could be helpful to understand rare disease mechanisms in 2D cell culture. These results should then be translated into suitable biofabricated or disease-on-a-chip systems to further increase their accuracy and reliability.

Although the research in engineered models of rare diseases is at its preliminary state, there have already been successful pilot studies. For instance, various multi-organ-on-a-chip platforms based on iPSCs and CRISPR technology have been proposed to induce specific mutations in an engineered cell population to study the autoimmune response and to understand the complex mechanism of autoimmune diseases, such as type I diabetes, rheumatoid arthritis, and celiac disease (de Mello et al., 2019). Since the severity of autoimmune diseases varies considerably amongst individuals, the potential to model combinations of genetic, environmental, and cellular components makes multi-organ-on-a-chip platforms uniquely able to determine the most relevant factors in disease progression for specific patients. For rare autoimmune diseases, where the incidence is too low to gather essential data on affected individuals and shared risk factors, multi-organ-on-a-chip systems can potentially be engineered with modules representing a patient’s own somatic and immune cells to determine the disease cause and to identify or test relevant therapeutics as a precision medicine application.

Along with the multi-organ-on-a-chip studies for autoimmune diseases, rare multifocal motor neuropathies have also been recently studied to understand the disease mechanisms at the cellular level. Hence, a human-based neuromuscular junction (NMJ) model has been suggested for congenital myasthenic syndrome, which commonly arises from mutations in one of the acetylcholine receptors encoding genes. The described 3D co-culture model provided a robust method to investigate adult human NMJ development and, for the first time, the adult forms of neuromuscular diseases *in vitro* (Bakooshli et al., 2019). Indeed, the use of engineered rare disease models is still in its infancy; nevertheless, the premise of these platforms has already started to play a pivotal role in streamlining the precision medicine processes.

#### Neurodegenerative Diseases

Neurodegeneration, the slow and progressive dysfunction and loss of neurons and axons in the central nervous system, is the primary pathological feature of acute and chronic neurodegenerative conditions. As neurodegeneration may occur due to genetic or phenotypic idiosyncrasies, it is of great interest in precision medicine. Although the neurodegenerative diseases may vary significantly in their clinical and pathological characteristics, at the molecular level, they share a fundamental pathogenic mechanism termed the seeded aggregation of disease-specific proteins. TDP-43 protein accumulation in amyotrophic lateral sclerosis, beta-amyloid accumulation in Alzheimer’s disease, and huntingtin protein accumulation in Huntingtin disease can be considered as the most common examples (Wegorzewska and Baloh, 2011; Park et al., 2015; Virlogeux et al., 2018). Alongside identifying disease-related protein accumulation, the interaction between the neurons and vascular cells has been established as a critical player in regulating the neuronal function and fate of neurodegenerative disease. Despite the recent progress—i.e., discovering the mutations, misfolded proteins, and neurovascular interaction—most of the human neurodegenerative conditions remain poorly understood, as the currently available models based on the transgenic mouse, rat, and non-human primate models, are unsatisfactory to reveal the interplay of genetic and environmental factors in many cases. Thus, 3D-biofabricated and disease-on-a-chip systems have been proposed as alternatives to create more realistic human neurodegenerative disease models. Recent studies have shown that by employing these systems, it is possible to obtain results comparable with the current gold-standard *in vivo* and 2D *in vitro* disease models. In few cases, these new platforms have even outperformed such standards, identifying the synergetic effects of genetic and environmental factors, neuroinflammation, and production of proinflammatory cytokines. For instance, the most recent achievements in the design of neurodegenerative disease models are the development of neural network-on-a-chip models and a corticostriatal network-on-a-chip for studying Alzheimer’s disease, Huntingtin disease, and amyotrophic lateral sclerosis (Wegorzewska and Baloh, 2011; Park et al., 2015; Virlogeux et al., 2018). Without a doubt, these platforms cannot recreate the higher-level cognitive processing of a human brain or the full impact of neurodegeneration on the body. Yet, these tunable platforms hold great potential in precision medicine for deciphering the molecular and cellular neuro-pathologies by integrating genetic and phenotypic idiosyncrasies into the disease models.

### Drug Development and Testing Drug Kinetics

The 1990s can be considered a golden era in the pharmaceutical industry as the discovery of several blockbuster drugs generated maximal profits for the pharmaceutical sector. However, in the last two decades, the increased regulatory scrutiny in drug safety has decelerated the fast growth resulting in reduced revenues (Krumholz et al., 2007). Moreover, key patent expirations between 2010 and 2014 caused a further decline in product proceeds, which reduced the budget spent on drug development (Khanna, 2012). The reduced R&D expenditures have tumbled the discovery of potential revenue-generating innovative drugs, creating a financially vicious circle.

In recent years, reports from regulatory agencies have indicated that only less than 7% of phase I drugs have been launched to market. Hence, the pharmaceutical industry has taken significant measures to increase the bench-to-market turnover of phase I drugs. Low bench-to-market turnover and high cost of the clinical trials have diverted pharmaceutical research into a more precise and accurate assessment of new drugs at the pre-clinical stage (Paul et al., 2010). Therefore, extensive efforts have been put into developing new screening methodologies for the pre-clinical stage, mimicking the reality of clinical trials alongside drug discovery. Thus, only the safest and most efficient new drugs will be qualified for clinical trials in a fast and financially feasible manner.

Drug safety is the primary concern of clinical drug attrition. Unfortunately, at the pre-clinical stage, overlooking the population variance and uncorrelated *in vivo* toxicity may jeopardize the drug’s success during clinical trials. At the clinical trial period of a new drug, ADME (absorption, distribution, metabolism, and excretion) profiles, dosing, side effects, toxicity, and interaction with different drugs should be evaluated considering the population variability. 3D biofabrication and organ-on-a-chip systems are already employed for quasi-*in vivo* toxicity testing subsidiary to animal studies. Alongside their use in toxicity testing, the pharmaceutical industry, ethics committees, and lawmakers have also started to debate whether these systems can be better predictive models to explore therapeutic efficiency (Ingber, 2020). Whereas 3D biofabrication approaches aim to contribute to drug testing research, up to date, organ-on-a-chip technologies have been mostly applied and even commercialized for toxicology testing at the pre-clinical level. Parallel to the benchwork, these systems have also been proposed for designing precise and accurate data sets for *in silico* libraries as machine learning became widely used in the field of computer-aided drug discovery and development of new drugs (Rifaioglu et al., 2019).

#### Quasi-*In Vivo* Toxicity Testing

The current pre-clinical *in vivo* testing is inadequate to satisfy population diversity in the majority of the cases, and often the absence of toxicity in animal models, do not correlate with a similar lack of toxicity in humans (Bailey and Balls, 2019). Up to date, different organ-on-a-chip devices and 3D-biofabricated tissue models such as blood vessels (Grigoryan et al., 2019), bone marrow (Chou et al., 2020), the gut epithelium (Madden et al., 2018), lung (Chou et al., 2020), liver (Nguyen et al., 2016), ocular compartment (Achberger et al., 2019), kidney epithelium (Homan et al., 2016), skin (Madden et al., 2018), and reproductive organ (Xiao et al., 2017) have been reported to recapitulate the structural and functional complexity of human organs to study tissue-specific toxicity and overcome the drawbacks in the animal testing. One of the main advantages of organ-on-a-chip systems is the possibility of using human cells derived from different donors to attain toxicity information related to diverse human populations. Thanks to such developments, some commercialized tissue-specific organ-on-a-chip devices have even started to become a standardized procedure for toxicity testing for several research groups. Indeed, designing functional tissue models to investigate organ-specific toxicity of drugs is beneficial; nonetheless, organ-specific requirements are not sufficient to provide a full understanding of drug safety. Thus, the proposed systems should enable the incorporation of fundamental physiological responses regarding ADME profiles, immunity, endocrinal effects, gut-microbiome interactions, effects on reproductive organs, and real-world multi pathology. Therefore, multi-organ-on-a-chip systems have been widely investigated, aiming at validating the functionality of each organ in the multi-organ-on-a-chip separately. Without a doubt, up to date, these studies have demonstrated, on the one hand, a high potential for the use of functionally coupled multi-organ-on-a-chip for drug assessments and, on the other hand, that highlighting the pertinent need to overcome existing challenges. First, these systems cannot be considered as an exact mimic of the entire *in vivo* effects but as a more predictive and biologically relevant assay for the drug discovery cascade. The main challenges in integrating organ-on-a-chips into multi-organ-on-a-chips are the optimization of custom cell culture medium formulations for each organ and developing of perfusion of the nutrients and the oxygen throughout the overall multi-organ-on-a-chip. Concurrently, thorough assessments in proper scaling of organ-on-a-chip models, implementation of real-time evaluation, acquiring renewable cell sources, developing chemical, mechanical, and electrical cues for missing organ systems, structural and functional validation of multi-organ-on-a-chip platforms required for the translation of these systems.

#### A New Avenue to Explore Therapeutic Efficiency

Despite the *in vivo* testing is currently the gold standard to explore therapeutic efficiency, qualitative and quantitative high-resolution analysis of diverse biological processes has not been possible in animal models. Thus, the demand for 3D-biofabricated tissues and organ-on-a-chip devices is significantly increasing thanks to their key advantages of enabling direct real-time or end-point analysis. These systems can reveal the collective and independent interaction of an organ’s different tissue components with new therapeutics and unveil the mechanism of drug action and their efficacy at the molecular level (Benam et al., 2016; Knowlton and Tasoglu, 2016; Srivastava et al., 2016; Song et al., 2020; Si et al., 2021). However, small culture volumes and low cell numbers in these platforms often give rise to technical issues associated with detection sensitivity and specificity. Research in the field is trying to overcome these technical issues by finding a focal point between what needs to be measured—the biomarkers—and which analytical method should be used in the detection limits for high specificity and sensitivity (Li and Tian, 2018). Biomarkers are specific molecules that can be objectively measured and evaluated as an indicator of a normal biological process, a pathological process, or a biological response to a therapeutic intervention. Precise identification of application-specific biomarkers is crucially important; hence, the collection and handling of all samples and all test performance should be conducted in a standardized manner. Therefore, analytical techniques, such as enzyme-linked immunosorbent assays (ELISA) (Luan et al., 2017; Gjorevski et al., 2020), liquid chromatography coupled with mass spectrometry (LC-MS) (Mao et al., 2018), and polymerase chain reactions (PCR) (Phan et al., 2017) as well as micro-total analysis systems (µTAS) (Fantuzzo et al., 2018), have been integrated within organ-on-a-chip platforms, showing high-resolution biochemical analysis for commercialized drugs and new therapeutic agents with substantially reduced sample volume requirements (Yesil-Celiktas et al., 2018).

#### Designing Precise and Accurate Data Sets for *In Silico* Libraries

Since the quantitative structure-activity relationship (QSAR) concept has been proposed back in the 1960s, QSAR-based models have been used in the lead-optimization step of drug discovery to assess various drug properties such as enzymatic reactions of drugs, drug-target interactions, and drug toxicity (Marx et al., 2020). Prospering and clinically coherent predictions of *in silico* tools have substantially contributed to drug screening and identifying potential new drug leads. Moreover, recently, with the integration of machine learning methods to develop QSAR models, extraction of non-parametric and non-linear relationships from datasets has been expedited. Consequently, these developments have accelerated the analysis of ineffective compounds and enabled the development of *in silico* models with better predictive performance. Indeed, the increased predictive values will serve for patient benefit and the reduction of *in vivo* testing. A predictive model’s power strongly relies on experimental data, and obtaining reliable experimental datasets can only be possible through automated and standardized experimental protocols. In this context, organ-on-a-chip platforms are extensively trying to meet machine learning platforms’ needs at all levels of their life cycle, training, and feedback loops. Ultimately, these systems are envisioned to create *in silico* libraries and a new level of bio-virtuality by bridging human *in vitro* and *in silico* models.

### The Convergence With Omic Analyses

Omic sciences attempt to comprehensively study the complex interactions between molecules in the different systems biology layers. With the progress and development of new postgenomic technologies, omics studies are becoming increasingly prevalent and more accessible to diverse disciplines. In order to succeed in the omic investigation, the correct design of the experimental part is crucial. Omic approaches, based on a holistic view of molecules, can improve the validity of preclinical predictions for drug response, which is essential to patient survival, and at the same time would reduce the cost of clinical practices. The existing technologies are differentiated by the specific target they will detect, and the leading omic techniques can be classified as genomics, transcriptomics, proteomics, and metabolomics. Whereas the analysis of gene expressions is the primary concern of genomics, the entire set of transcripts, including coding and non-coding RNAs, are the subjects of transcriptomics. Additionally, proteomics and metabolomics are involved in investigating the whole set of proteins and the large-scale study of metabolites of a cell, tissue, or organ.

The modern evolution of omic practices has brought the resolution of analysis at the single-cell level. The refinement of technologies has moved the challenge towards studying small quantities of molecules contained in each cell (Chappell et al., 2018). To date, omic analyses have allowed us to reconstruct networks and pathways that have been used to extrapolate the functional interactions between genes, proteins, and metabolites. Furthermore, single-cell approaches have highlighted the heterogeneity among cells within populations previously considered homogeneous (Vemuri and Aristidou, 2005; Bodein et al., 2020; Xing et al., 2020). Unfortunately, many preclinical omic data were generated within 2D biological systems and thus are affected by the lack of contribution of the extracellular microenvironment, the parenchymal and vascular compartments, and the tissue-tissue interface. Therefore, only a few of them have generated new algorithms with an adequate prognostic capacity to be used in medical practices (Sontheimer-Phelps et al., 2019; Canzler et al., 2020). Consequently, the combined use of high-throughput omic methods with 3D biofabrication or organ-on-a-chip technologies could represent an opportunity to validate new drug targets and develop new personalized therapeutic strategies.

As mentioned above, several *in vitro* organ and 3D tumor models have already been generated, and their deep characterizations are progressively implemented by emerging multi-omic technologies (Agarwal et al., 2017; Trujillo-de Santiago et al., 2019). However, to date, mostly microtissues cultured within OOC systems have been used as inputs for omic analyses—which are generally performed off-chip—while no significant steps have been reported in the integration of biofabricated samples and omics approaches. In particular, OOCs coupled with omics have been developed based on the profiling of the metabolome in microfluidic bioartificial organs with the ability to identify toxicity markers *in vitro* (Shintu et al., 2012; Lee D. W. et al., 2017; Jellali et al., 2018). For example, Wang and colleagues created an excellent human organ-on-a-chip system integrating, for the first time, cell engineering as well as drug metabolism and metabolomics to imitate complex human physiology and multi-organ interconnections (Wang X. et al., 2019). With this system, the metabolomics of ‘*tolcapone,’* a medicine used to treat the symptoms of Parkinson’s disease, has been able to generate complete metabolite profiles, highly representative of the human system. More recently, Ndiaye *et al.* presented a ChipFilter Proteolysis (CFP) microfluidic platform as a proteomics bioreactor for the miniaturization of protein extraction steps for bottom-up proteomics approaches. This system allows the combination of a molecular filtration membrane in PDMS microchip, using soft lithography and replica molding, promoting efficient protein retention and proteolysis on the membrane, and reducing significantly the time for proteome analysis and the amount of the samples if compared with membrane-based commercial ultracentrifugation cartridges (Ndiaye et al., 2020).

Furthermore, integration with microfluidic platforms could further allow the assessment of *in situ* cytotoxicity and the dynamic drug-transport and -delivery behavior from nanocarriers in the same system (Zhang et al., 2017b; Chen et al., 2018, 2021). The dizzying expansion of these technologies has, however, seen the need to re-isolate the cells once they have done their job within a complex bioprinted or microfluidic system. To date, methods for isolating single-cells in a format compatible with single-cell omic experiments include laser acquisition microdissection (Emmert-Buck et al., 1996), manual micromanipulation (Hu et al., 2016), patch-clamp method (Bussard et al., 2016), and Raman tweezers (Snook et al., 2009). However, the high amount of work does not guarantee a high percentage of isolated cells used in the experimental system, sometimes tens and sometimes hundreds per study. Therefore, increasing the number of cells profiled is the right strategy for overcoming the noise that is intrinsic in single-cell measurements. In the past few years, microfluidics-based chips have been established to increase the number of cells profiled, reducing the experimental costs and coupling the tissue engineering approaches to the next-generation sequencing, a currently preferred method for omics analyses. To date, several microfluidic devices have been adapted to address concerns of productivity and cost in single-cell preparation and analysis. The main method couples microfluidic channels with pressure-control valves (Whitesides, 2006; Fan et al., 2011). Other innovative techniques for detecting single-cells and their components are based on using a microfluidic apparatus that is able to capture them in an inert carrier oil or using arrays of nanoliter-scale wells (nano-wells) in which, by gravity, cells or cellular components are seeded at low densities to reach a single element per well (Prakadan et al., 2017). Each of these approaches can be used to establish the interconnection between single cells, capture their specific products, and retain their components upon lysis. Importantly, given their small size, these features can be used to process many single cells in a compact physical space, reducing reagent requirements (and thus costs) and increasing analyte concentrations and thus assay efficiency when limited kinetically or by background. These innovative systems that focus on cellular output once intercellular dynamics are exhausted, have significantly improved the performance of single-cell omic studies, enabling the parallel processing of thousands of cells. The platforms developed so far not only exploit the experience of providing realistic physiological models, but also significantly contribute to reducing the costs for the preparation of molecular libraries for single-cell approaches by reducing the reaction volumes to the scale of nano/picoliters (Esch et al., 2015).

## Discussion

In the last two decades, biomedical research has greatly benefited from introducing highly sophisticated methods to fabricate more advanced *in vitro* models of tissues and organs. Organ-on-chips and biofabrication technologies are two great examples of them. These technologies are effectively driving a revolution in several areas of biomedical research. In this review, we have initially provided a brief overview of the broad set of available biofabrication and organ-on-a-chip technologies. This is followed by analyses of how they have been exploited by researchers in few research scenarios, which include the fabrication of functional, implantable tissue/organ substitutes, the development of platforms for modelling diseases or drug testing/screening, and the integration of omic analyses. As described in the text, the progress made is relevant; however, we are still far from translating these solutions into actual clinical scenarios due to unmet technical challenges and lack of fundamental knowledge of the biological processes involved (Hoffman et al., 2019).

In the context of tissue engineering and regenerative medicine, biofabrication technologies have affirmed themselves as the gold standard enabling to recapitulate architectural complexity and dimensions of human organs. The advantages offered by these technologies are numerous, including automated generation of 3D, biologically relevant tissues with high precision and repeatability. The 3D structures biofabricated so far, despite their realistic shapes and dimensions, still exhibit limited functionalities, thus being unsuitable directly for clinical applications. As one could easily infer, fabricating artificial tissues/organs is a highly complex task, and, up to date, it is still difficult to precisely identify and foresee all the actions required for this process. Typical current approximations entail the number and type of cells, their 3D spatial distribution, and the presence of a functional vasculature and innervation. The latter features are required to supply nutrients to each cell in the construct and, depending on the target tissue, support a proper integration with the host nervous system upon construct grafting *in vivo*. Among the possible challenges, those connected with the scaling-up processes to manufacture an idoneous number of cells will most likely need in the future a thorough revision and improvement to fulfil the stringent requirements of GMP production. Additionally, researchers will need to understand better the relations between bioink properties and the fate of embedded cells. So far, in fact, the focus has been mainly directed to formulate bioinks that would enable high printing resolution and support preliminary tissue maturation. Last but not least, on the bench to clinical translation side, designing mechanically stable, biocompatible, and financially feasible constructs can be considered the utmost concern.

Regarding the complex field of disease modelling, biofabricated constructs and OOC platforms significantly complement each other to develop an understanding of disease mechanisms and precision medicine. Indeed, biofabricated and OOC disease models are not a need or a unique tool for all diseases, and currently, these platforms are finding promising applications in some medical fields, such as cancer, neurodegenerative and rare diseases, to assure effective treatments and bench-to-bedside transition of these treatments for patient subgroups with similar epigenetic profiles. For instance, tumor-on-chip platforms have taken particular attention in pediatric oncology, where they could be used to test tissues harvested from patients as an alternative to risky first-in-human studies within pediatric populations. Alternatively, on the rare disease front, pioneering models for rare disorders, e.g., Barth syndrome, progeria, Timothy syndrome, and hereditary hemorrhagic telangiectasia, cystic fibrosis, have been suggested for clinical trial implementation and therapeutic development (Wang et al., 2014; Hinson et al., 2015; Atchison et al., 2017; Ribas et al., 2017; Shik Mun et al., 2019). Moreover, since the outbreak of the COVID-19 pandemic, such platforms have also been exploited to understand the effects of the SARS-CoV-2 virus on respiratory tissues, underlining once again their great potential (Si et al., 2021; Zhang et al., 2021). Overall, biofabricated and OOC disease modeling platforms are still facing some challenges with their integration into clinical applications. From the biomimicry perspective, embodying immune and endocrine responses in such platforms is still a significantly complex and daunting task. Moreover, physiochemically relevant disease modeling platforms that are standardized and user-friendly have not been fully achieved yet, apart from few commercialized examples (Nawroth et al., 2020; Apostolou et al., 2021; Fengler et al., 2021; Vormann et al., 2021).

In drug development, surely, OOC platforms and biofabricated tissues cannot be considered as an exact mimic of the entire *in vivo* effects but as a more predictive and biologically relevant assay for the drug discovery cascade. More specifically, to understand and evaluate the efficacy of the drugs and their ADME profile, developing multi-organ systems—i.e., miniaturized chips embedding various organ/tissue-specific compartments—has become a necessary requirement. The main challenges in developing such multi-organ platforms entail optimizing universal cell culture medium formulations to simultaneously support all organ/tissue compartments, acquiring renewable cell sources, and developing biomimetic perfusion of the nutrients/oxygen throughout the whole system. Additionally, to achieve a structurally and functionally validated multi-organ-on-a-chips, a thorough assessments in proper scaling of organ-on-a-chip models, implementation of real-time evaluation, and development of chemical, mechanical, and electrical cues for missing organ systems stand as crucial requirements.

Finally, the combined use—although off-chip—of omic approaches with OOCs has led to new insights in providing realistic physiological models and significantly reducing the costs for the preparation of molecular libraries for single-cell approaches by reducing the reaction volumes to the scale of nano/picoliters. These innovative systems that focus on cellular output have significantly improved the performance of single-cell omic studies, enabling the parallel processing of thousands of cells cultured in such a tailored microenvironments.

All in all, OOC platforms and biofabrication systems are largely considered the way-to-go strategies to develop advance tissue/organ models and, most likely, will continue to play a key role in the next decades.

## Future Outlook

Undoubtedly, in the last few years, biofabrication and organ-on-a-chip strategies have had a significant, positive impact on biomedical research through the fabrication of increasingly refined 3D tissue/organ models. While these first successes are a matter of fact, it is not trivial to foresee how these technologies will further develop over the next one or two decades and how their outputs will be eventually translated from the realm of research to meaningful clinical applications. Nevertheless, these systems are nowadays affected by some common limitations which should be necessarily addressed in the near future. Given the inherent complexity of advanced, *in vitro* tissue model manufacturing, these limits do not have clear boundaries, being often intertwined one to the other.

First, the variability of the experiments among different batches and systems should be drastically reduced through a thorough standardization of the whole process. Both biofabrication and organ-on-a-chip systems, in fact, lack any specific guideline that should help researchers in developing regulatory-approvable products and, only recently have researchers started to define common roadmaps to address standardization issues (Mastrangeli et al., 2019a; Sun et al., 2020). Such standardization should affect all the aspects of these biotech strategies, including material and cell selection, isolation and purification, bio-construct manufacturing, processing and characterizations, culturing protocols, and bio-construct post-processing. The first step towards this direction has been lately made by the United States Food and Drug Administration (FDA) in its predictive toxicology roadmap where organ-on-a-chip models have been identified as new promising approaches to develop innovative toxicology methods and have been adopted in FDA laboratories to assess their capacities (Food and Drug Administration, 2017). As pointed out in the FDA document, the acceptance of any new methods will require sufficient convincing data as well as continuous dialogue and feedback among all relevant stakeholders from development to implementation, including, in particular, validation and acceptance by regulatory authorities. Reasonably, the implementation of organ-on-a-chip platforms by a regulatory agency should promote and accelerate standardization of this technology at different levels.

The second challenge that should be addressed in the near future consists of the increase in the reliability and robustness of the manufactured models. These aspects represent a key point for the adoption of these technologies in clinically relevant contexts. To improve tissue model reliability and robustness, a more thorough and deeper characterization of the inputs, outputs, and models themselves and their cross-impacts will be needed. This will require the simultaneous analysis of tens up to hundreds of variables/parameters at the same time with the generation of extremely large volumes of data. Performing data analytics of such voluminous data sets is generally complex, and new *in silico* tools should be specifically developed in collaboration with mathematicians, statisticians, and bioinformaticians. Big data analysis, deep learning, and artificial intelligence methods have already been effectively applied and implemented in many industrial and research fields and technologies, and it is reasonable to foresee that soon they might also be integrated with the most advanced biotech systems for organ/tissue modeling (Galan et al., 2020; Xu and Ye, 2020; Zhou et al., 2020). Of note, generating these datasets, at least in the near future, is expected to be time-consuming and costly and, therefore, impractical at the single laboratory or small start-up levels. A possible solution to this economic problem could be establishing research clusters/networks where scientists could tackle this challenge through a common effort.

The third issue that affects both current biofabrication and organ-on-a-chip models is their oversimplified nature and thus poor functional behavior, primarily due to persisting technological limitations and lack of fundamental knowledge. By definition, a model is a simplified version of the actual tissue/organ found *in vivo*. However, as demonstrated in numerous studies, a proper recapitulation of the tissue microenvironment is essential to obtain *in vitro* functionalities comparable to native tissues/organs. To this end, the main focus of the research community had been to recreate static microenvironments, which are still far from dynamic native ones. In this regard, recently, the integration of 3D printing technology and smart shape-memory materials has created a great wave of enthusiasm for developing physiologically more relevant tissue models, establishing a new research field termed 4D printing. With the introduction of a fourth dimension, *i.e.*, ‘time,’ the 4D printing technology allows both spatial and temporal control over the fabricated constructs, better mimicking dynamic tissue responses towards certain natural stimuli (Tamay et al., 2019; Hann et al., 2020). Though this technology is still in its infancy, it has already succeeded in placing a landmark in the sphere of biomedical research, holding promising prospects for further advancements in the near future.

Besides vascularization, innervation, spatially defined cell distribution, ECM composition, biochemical and electro-mechanical stimulation, and inter-organ cross-talks still remain as pertinent challenges and are hardly observed in the currently available biofabricated or organ-on-a-chip models. In order to overcome these limits, a great deal of work will surely be needed in different research domains, ranging from biomaterials and biochemistry to bioengineering, cell and developmental biology, and bioinformatics.

Finally, it is foreseen for the near future convergence of these two sets of technologies that would eventually enable the manufacturing of cellularized OOC systems using additive manufacturing platforms (Knowlton et al., 2016; Yi et al., 2017; Mi et al., 2018a; Carvalho et al., 2021). To date, this research area—i.e., 3D printing of microfluidic devices—is growing at an impressive pace following the tremendous technological advancements in 3D printing, which enable to simultaneously manipulate multiple materials (bio- and non-bio materials) at high printing resolution. Specifically, researchers have developed few strategies to combine OOC and 3D bioprinting: 1) the use of 3D printing systems to manufacture structures for OOC replica molding, 2) bioprinting of micro-tissues within pre-fabricated OOC, and 3) one-step fabrication of the cellularized OOC. The convergence of these technologies is expected to merge their intrinsic advantages, thus providing more customizable and controllable microenvironments for the bioprinted tissues, which should eventually lead to more realistic tissue/organ models. However, there is still some work ahead—especially for the more attractive case of one-step fabrication of cellularized OOC—due to some material (for instance, the optical properties of the printed OOC are still unsatisfactory) and technological limitations.

## Conclusion

In conclusion, biofabrication and organ-on-a-chip methods represent the most promising technologies nowadays available to advance biomedical research and clearly will play a key role in academic and industrial research during the next two decades. As pointed out in this review, these technologies have already found applications in almost all branches of biomedical research, being also the catalyst for the establishment of as many promising start-up companies. Roadmaps have been set to guide the future development of both research fields, and, hopefully, regulatory agencies worldwide will soon accelerate the redaction of guidelines to implement these systems into more relevant clinical scenarios.
